# Distributional patterns of item responses and total scores of the Patient Health Questionnaire for Adolescents in a general population sample of adolescents in Japan

**DOI:** 10.1111/pcn.13148

**Published:** 2020-09-29

**Authors:** Masaki Adachi, Michio Takahashi, Tomoya Hirota, Hiroki Shinkawa, Hiroyuki Mori, Takuya Saito, Kazuhiko Nakamura

**Affiliations:** ^1^ Department of Clinical Psychological Science Graduate School of Health Sciences Hirosaki Japan; ^2^ Research Center for Child Mental Development, Graduate School of Medicine Hirosaki University Hirosaki Japan; ^3^ Department of Psychiatry University of California, San Francisco San Francisco USA; ^4^ Department of Child and Adolescent Psychiatry, Graduate School of Medicine Hokkaido University Sapporo Japan; ^5^ Department of Neuropsychiatry, Graduate School of Medicine Hirosaki University Hirosaki Japan

Major depression is one of the most prevalent mental health problems in adolescents and is associated with increased risk for subsequent attempted or completed suicide,^1^ comprising more than half of the reported adolescent suicide victims at death.^2^ Nevertheless, adolescent depression is more frequently missed than that in adults, possibly due to the prominence of irritability, mood reactivity, and fluctuating symptoms.^3^ For the early detection of depression, screening scales are clinically important; therefore, the optimal cut‐off score to detect major depressive disorder in various populations has been investigated.^4^ However, distributional patterns of item responses in the adolescent general population are limited. In order to detect adolescent depression, which is often overlooked, the distributional pattern of depression symptoms in the general population should be deeply understood. The Patient Health Questionnaire for Adolescents (PHQ‐A) is a self‐reporting questionnaire adapted from the adult version of the PHQ‐9, one of the most used screening tools for major depression worldwide.^4^ The PHQ‐A is used to screen for depression among adolescents in a developmentally appropriate fashion in accordance with the DSM‐IV‐TR criteria.^5^ As nine components of the PHQ‐A match the ‘A’ diagnostic criteria for major depression in the DSM‐5,^6^ the distributional pattern of PHQ‐A item responses in the adolescent general population should be examined to deepen our understanding of various expressions of depression symptoms among adolescents. Therefore, this study provided distributional patterns of item responses and total scores using the PHQ‐A among adolescents.

Every year, a community‐based school survey has been prospectively conducted to measure several mental health indicators and their associated factors among adolescents in Hirosaki (please see Appendix S1 for more information about Hirosaki City).^7^ Data were obtained in September 2019 targeting children between the 4th and 9th grades in public elementary and junior high schools. We distributed 8003 sets of the PHQ‐A to the corresponding schools. Classroom teachers explained the contents of this survey and discussed the concept of informed consent with them. Additionally, we mailed letters and information on the study to each child's primary caregiver(s), and we excluded the children whose primary caregivers indicated an intention of not wanting their children to participate; the cohort included a total of 7765 children (3850 boys [49.6%] and 3915 girls [50.4%]). Ethical approval was obtained from the Hirosaki University Committee on Medical Ethics (IRB#2019‐1026). This study was conducted in accordance with the ethical standards laid down in the 1964 Declaration of Helsinki and its later amendments.

Before examining the distributional pattern of PHQ‐A item responses, the psychometric properties of the PHQ‐A for Japanese adolescents were confirmed, which have not been reported to date (please see Appendix S2).

Table 1 displays the PHQ‐A item responses. Item responses for all nine items showed a similar pattern. Such a distributional pattern was also reported in a PHQ‐9 study in adults.^8^ The distribution of No. 9 (suicidal ideation) showed a difference between current and previous studies conducted on adult participants^8^: 83.5% and 96.6% for *not at all*, 10.7% and 15.0% for *several days*, 3.1% and 0.6% for *more than half the days*, and 2.4% and 0.6% for *nearly every day*, respectively. This difference was statistically significant (χ^2^ = 530.1, d.f. = 3, *P* < 0.001), suggesting that adolescents may be more frequently prone to suicidal ideation and suicide attempts than adults. This result is considered to reflect the current situation in Japan where the suicide rate for adults is decreasing; however, those for children and adolescents remain high.^9^

**Table 1:** Distributional patterns of the Patient Health Questionnaire for Adolescents item responses (*n* = 7765)

	Not at all		Several days		More than half the days		Nearly every day		Missing	
Statement	*n*	%	*n*	%	*n*	%	*n*	%	*n*	%
Feeling down, depressed, irritable, or hopeless?	4909	63.2	2189	28.2	400	5.2	251	3.2	16	0.2
Little interest or pleasure in doing things?	5552	71.5	1613	20.8	348	4.5	210	2.7	42	0.5
Trouble falling asleep, staying asleep, or sleeping too much?	4179	53.8	1976	25.4	758	9.8	824	10.6	28	0.4
Poor appetite, weight loss, or overeating?	5226	67.3	1564	20.1	557	7.2	384	4.9	34	0.4
Feeling tired or having little energy?	3702	47.7	2476	31.9	891	11.5	679	8.7	17	0.2
Feeling bad about yourself, or feeling that you are a failure, or that you have let yourself or your family down?	4954	63.8	1625	20.9	592	7.6	573	7.4	21	0.3
Trouble concentrating on things like schoolwork, reading, or watching TV?	5631	72.5	1364	17.6	426	5.5	315	4.1	29	0.4
Moving or speaking so slowly that other people could have noticed? Or the opposite – being so fidgety or restless that you were moving around a lot more than usual?	6295	81.1	1035	13.3	254	3.3	160	2.1	21	0.3
Thoughts that you would be better off dead, or of hurting yourself in some way?	6481	83.5	832	10.7	243	3.1	188	2.4	21	0.3
Average	5214	67.2	1630	21.0	496.6	6.4	398.2	5.1	25.44	0.3

Regarding the PHQ‐A total scores, 21.6% of participants had a score of 0, whereas 63.4% of them had a score of 0–4; the percentage of participants who scored 10, which is the cut‐off score of the PHQ‐9,^4^ was 11.1% (please see Appendices [Link], [Link]). These severity distributions are overall consistent with findings reported in the National Comorbidity Survey – Adolescent Supplement, which reported the prevalence of depression in adolescents and revealed that approximately 11% of adolescents have a depressive disorder by age 18 years.^10^

This study presents distributional patterns of PHQ‐A item responses and total scores in the Japanese adolescent general population, which have not been reported previously. Although study limitations cannot be ignored (e.g., a single‐area study; no data are available for students who were absent from school during the survey period; the discriminant validity, including the cut‐off score of the PHQ‐A, has not been examined), this study has several strengths. Targeting all children in public elementary and junior high schools in one area with a high participation rate (97.0%) yields highly relevant community‐based data. These data are the baseline for the long‐term trajectory of depressive symptoms in our ongoing prospective cohort study. In future studies, the heterogeneity of the developmental trajectory should be determined among these nine symptoms, that is, the DSM‐5 ‘A’ diagnostic criteria for major depression.

## Disclosure statement

The authors declare that they have no competing interests.

## Supporting information


**Appendix S1.** Information about Hirosaki City.

**Appendix S2.** Psychometric properties of the Patient Health Questionnaire for Adolescents for Japanese adolescents.

**Appendix S3.** Distributional patterns of the Patient Health Questionnaire for Adolescents total scores.

**Appendix S4.** The Patient Health Questionnaire for Adolescents total score and severity classification (*N* = 7612).
