# Supplementary material for: Distributional patterns of item responses and total scores of the Patient Health Questionnaire for Adolescents in a general population sample of adolescents in Japan
Source: Psychiatry Clin Neurosci. 2020 Sep 29;74(11):628–9. doi: 10.1111/pcn.13148 (PMC7702070; doi:10.1111/pcn.13148)
Supplement: Supplementary file 1 — Appendix S1. Information about Hirosaki City. [file PCN-74-628-s001.docx]

**Supporting Document 1: Information about Hirosaki City**

Hirosaki city is located in the northern part of Japan’s main island. This city is about 524.20 km2 in size, and the total population was reported as 174,171 in 71,823 households ^(1)^. The taxable annual income per taxpayer in Hirosaki City is 2,687,000 Yen, which is close to the national level (2,747,000 Yen) ^(2)^. Hirosaki City has 52 public elementary and junior high schools (35 elementary schools and 17 junior high schools), and there is only one private school (junior high school). About 99.4% of children are enrolled in public elementary and junior high schools.

The suicide rate in Hirosaki City tended to be higher than the national statistics from 2007 to 2009, but since 2010 it has been at the same or lower level than the national level. According to the latest data for 2019, the suicide rate in Japan was 15.8% for a population of 100,000, while the suicide rate in Hirosaki City was 14.0% ^(3-4)^.

**References for Supporting Document 1**

1. The Statistics Bureau of Japan. Population Census [Internet]. [cited 2020 Jan 31]. Available from: https://www.e-stat.go.jp/en/stat-search/files?page=1&layout=datalist&toukei=00200521&tstat=000001080615&cycle=0&tclass1=000001089055&tclass2=000001089057&tclass3=000001089059&stat_infid=000031473346&second2=1

2. Cabinet office: Population and economic data by municipality [Internet]. [cited 2020 July 20]. Available from: https://www5.cao.go.jp/keizai-shimon/kaigi/special/future/keizai-jinkou_data.html.

3. Hirosaki City: Hirosaki suicide countermeasure plan [Internet]. [cited 2020 July 21]. Available from: http://www.city.hirosaki.aomori.jp/jouhou/keikaku/files/20190716all.pdf

4. Ministry of Health, Labour and Welfare: Suicide Statistics: Basic material for suicide in the community [Internet]. [cited 2020 July 21]. Available from: https://www.mhlw.go.jp/stf/seisakunitsuite/bunya/0000140901.html
