# Supplementary material for: Distributional patterns of item responses and total scores of the Patient Health Questionnaire for Adolescents in a general population sample of adolescents in Japan
Source: Psychiatry Clin Neurosci. 2020 Sep 29;74(11):628–9. doi: 10.1111/pcn.13148 (PMC7702070; doi:10.1111/pcn.13148)
Supplement: Supplementary file 2 — Appendix S2. Psychometric properties of the Patient Health Questionnaire for Adolescents for Japanese adolescents. [file PCN-74-628-s002.docx]

**Supporting Document 2: Psychometric property of the Patient Health Questionnaire for Adolescents for Japanese.**

For factorial validity, the factor structure of the PHQ-A was examined with confirmatory factor analysis (CFA). The analysis was conducted by specifying the unidimensional model, which was reported in the previous study of PHQ-A (1). Models were estimated with mean and variance-adjusted weighted least squares estimation (WLSMV) for use with ordinal items (2). Model fit was evaluated using the chi-square value, the comparative fit index (CFI) and the root mean square error of approximation (RMSEA). A general consensus on acceptable levels of fit indices is a values of .95 or more for CFI and a value of .08 or less for RMSEA (3). For construct validity, we investigated PHQ-A scale intercorrelations with the 7-item Generalized Anxiety Disorder questionnaire (GAD-7), the KIDSCREEN-27. The GAD-7 is a one-dimensional instrument designed to detect symptoms of generalized anxiety disorder as it is defined in the DSM-IV. The item scores range from 0 (not at all) to 3 (nearly every day), resulting in a sum score range from 0 to 21 (4). The KIDSCREEN-27 assesses Health-related quality of life (HRQoL) in five dimensions: physical well-being (5 items); psychological well-being (7 items); autonomy & parents (7 items); social support & peers (4 items); and school environment (4 items). Responses to KIDSCREEN-27 questionnaire measure frequency (never-seldom-sometimes-often-always) or intensity (not at all-slightly-moderately-very-extremely) of the assessed attribute with the 5-options Likert scale, with a recall period of one week. Rasch scores are computed for each dimension and are transformed into T-values with a mean of 50 and a standard deviation of 10; higher scores indicate better HRQoL and well-being (5). Mplus 7 software was used for CFA and PASW Statistics 25 (SPSS) software was used for other analyses.

To evaluate the unidimensional structure of the PHQ-A, all nine items were specified as indicators of one factor. Factor loadings were generally high, ranging from .58 to .83. This model fit the data well, as indicated by robust fit indices CFI = .967, RMSEA = .077, and the 90% confidence interval for RMSEA = .074–.079. The intercorrelations between the PHQ-A total scale score and the GAD-7, the KIDSCREEN-27, were r = .74, r = −.64 (all p < .001), respectively. Additionally, the internal consistency of the entire PHQ-A using Cronbach’s alpha was also satisfactory (α = 0.84).

**References for Supporting Document 1**

1. Naveed S, Waqas A, Memon AR, Jabeen M, Sheikh MH. Cross-cultural validation of the Urdu translation of the Patient Health Questionnaire for Adolescents among children and adolescents at a Pakistani school. Public Health. 2019; **168**: 59-66.

2. Flora DB, Curran PJ. An empirical evaluation of alternative methods of estimation for confirmatory factor analysis with ordinal data. Psychol Methods. 2004; **9**: 466-91.

3. Hu LT, Bentler PM. Cutoff Criteria for Fit Indexes in Covariance Structure Analysis: Conventional Criteria Versus New Alternatives. Structural Equation Modeling-a Multidisciplinary Journal. 1999;6(1):1-55.

4. Spitzer RL, Kroenke K, Williams JB, Lowe B. A brief measure for assessing generalized anxiety disorder: the GAD-7. Archives of internal medicine. 2006; **166**: 1092-7.

5. Ravens-Sieberer U, Auquier P, Erhart M, et al. The KIDSCREEN-27 quality of life measure for children and adolescents: psychometric results from a cross-cultural survey in 13 European countries. Qual Life Res. 2007; **16**: 1347-56.
