# Supplementary material for: Distributional patterns of item responses and total scores of the Patient Health Questionnaire for Adolescents in a general population sample of adolescents in Japan
Source: Psychiatry Clin Neurosci. 2020 Sep 29;74(11):628–9. doi: 10.1111/pcn.13148 (PMC7702070; doi:10.1111/pcn.13148)
Supplement: Supplementary file 3 — Appendix S3. Distributional patterns of the Patient Health Questionnaire for Adolescents total scores. [file PCN-74-628-s003.docx]

| Severity | Score | Number | Percent | Cum percent |
| --- | --- | --- | --- | --- |
| No or Minimal depression | 0 | 1644 | 21.6 | 21.6 |
|  | 1 | 982 | 12.9 | 34.5 |
|  | 2 | 898 | 11.8 | 46.3 |
|  | 3 | 721 | 9.5 | 55.8 |
|  | 4 | 583 | 7.7 | 63.4 |
| Mild depression | 5 | 461 | 6.1 | 69.5 |
|  | 6 | 422 | 5.5 | 75.0 |
|  | 7 | 344 | 4.5 | 79.5 |
|  | 8 | 266 | 3.5 | 83.0 |
|  | 9 | 259 | 3.4 | 86.4 |
| Moderate depression | **10** | **186** | **2.4** | **88.9** |
|  | 11 | 168 | 2.2 | 91.1 |
|  | 12 | 123 | 1.6 | 92.7 |
|  | 13 | 98 | 1.3 | 94.0 |
|  | 14 | 84 | 1.1 | 95.1 |
| Moderately severe depression | 15 | 79 | 1 | 96.1 |
|  | 16 | 79 | 1 | 97.2 |
|  | 17 | 37 | 0.5 | 97.7 |
|  | 18 | 33 | 0.4 | 98.1 |
|  | 19 | 32 | 0.4 | 98.5 |
| Severe depression | 20 | 42 | 0.6 | 99.1 |
|  | 21 | 14 | 0.2 | 99.3 |
|  | 22 | 12 | 0.2 | 99.4 |
|  | 23 | 10 | 0.1 | 99.5 |
|  | 24 | 14 | 0.2 | 99.7 |
|  | 25 | 1 | 0.0 | 99.7 |
|  | 26 | 5 | 0.1 | 99.8 |
|  | 27 | 15 | 0.2 | 100.0 |

**Supporting Document 3: Distributional patterns of total scores the Patient Health Questionnaire for Adolescents.**
