# Supplementary material for: Distributional patterns of item responses and total scores of the Patient Health Questionnaire for Adolescents in a general population sample of adolescents in Japan
Source: Psychiatry Clin Neurosci. 2020 Sep 29;74(11):628–9. doi: 10.1111/pcn.13148 (PMC7702070; doi:10.1111/pcn.13148)
Supplement: Supplementary file 4 — Appendix S4. The Patient Health Questionnaire for Adolescents total score and severity classification (N = 7612). [file PCN-74-628-s004.docx]

**Supporting Document 4:** **The Patient Health Questionnaire for Adolescents total score and severity classification (*N* = 7612)**

|  | Severity distribution | |  |  |  |  | PHQ-A score | |  |
| --- | --- | --- | --- | --- | --- | --- | --- | --- | --- |
|  | No or Minimal | Mild | Moderate | Moderately Severe | Severe |  | Total | Male | Female |
|  | *n* [%] | *n* [%] | *n* [%] | *n* [%] | *n* [%] |  | *M* (SD) | *M* (SD) | *M* (SD) |
| Grade (age) |  |  |  |  |  |  |  |  |  |
| Primary school |  |  |  |  |  |  |  |  |  |
| 4 (9-10) | 750 | 273 | 129 | 39 | 19 |  | 4.5 (5.0) | 4.9 (5.2) | 4.1 (4.7) |
| n = 1210 | [62.0%] | [22.6%] | [10.7%] | [3.2%] | [1.6%] |  |  | n = 610 | n = 600 |
| 5 (10-11) | 826 | 261 | 84 | 38 | 14 |  | 3.9 (4.5) | 4.3 (4.8) | 3.7 (4.2) |
| n =1223 | [67.5%] | [21.3%] | [6.9%] | [3.1%] | [1.1%] |  |  | n = 605 | n =618 |
| 6 (11-12) | 850 | 257 | 80 | 36 | 17 |  | 3.9 (4.6) | 4.1 (4.7) | 3.7 (4.4) |
| n = 1240 | [68.5%] | [20.7%] | [6.5%] | [2.9%] | [1.4%] |  |  | n =639 | n = 601 |
| Junior high school |  |  |  |  |  |  |  |  |  |
| 7 (12-13) | 801 | 316 | 124 | 52 | 20 |  | 4.6 (4.8) | 4.6 (4.8) | 4.6 (4.8) |
| n = 1313 | [61.0%] | [24.1%] | [9.4%] | [4.0%] | [1.5%] |  |  | n = 647 | n = 666 |
| 8 (13-14) | 853 | 290 | 107 | 44 | 16 |  | 4.3 (4.6) | 3.8 (4.4) | 4.8 (4.8) |
| n = 1310 | [65.1%] | [22.1%] | [8.2%] | [3.4%] | [1.2%] |  |  | n = 657 | n = 653 |
| 9 (14-15) | 748 | 355 | 135 | 51 | 27 |  | 5.0 (5.0) | 4.3 (4.6) | 5.6 (5.2) |
| n = 1316 | [56.8%] | [27.0%] | [10.3%] | [3.9%] | [2.1%] |  |  | n = 597 | n = 719 |
|  |  |  |  | ANOVA for PHQ-A score | |  | *F* | *p* | *η_p_^2^* |
|  |  |  |  | Scool grade |  |  | 9.5 | > .001 | .006 |
|  |  |  |  | Gender |  |  | 1.28 | .258 | > .001 |
|  |  |  |  | School grade × gender | |  | 10.89 | > .001 | .007 |
| Participants with missing values were excluded from this analysis (*n* = 153).  PHQ = Patient Health Questionnaire for Adolescents, ANOVA = analysis of covariance | | | | | | | | | |
